# Health-Related Quality of Life for Children and Adolescents with Specific Language Impairment: A Cohort Study by a Learning Disabilities Reference Center

**DOI:** 10.1371/journal.pone.0166541

**Published:** 2016-11-16

**Authors:** Gaëlle Hubert-Dibon, Marie Bru, Christèle Gras Le Guen, Elise Launay, Arnaud Roy

**Affiliations:** 1 Department of Pediatrics, University Hospital Nantes, Nantes, France; 2 Learning Disabilities Reference Center, University Hospital Nantes, Nantes, France; 3 Psychology Laboratory of Pays de la Loire, Angers University, Angers, France; IRCCS E. Medea, ITALY

## Abstract

**Objectives:**

To assess the health-related quality of life (HRQOL) of children with specific language impairment (SLI).

**Study Design:**

In a prospective sample at a Learning Disabilities Reference Center, proxy-rated HRQOL (KIDSCREEN-27) was assessed for children with SLI and unaffected children from January 1, 2014 to March 31, 2015. Quality of life predictors for children with SLI were evaluated by recording the length and number of speech therapy and psychotherapy sessions and the specific school organization that the children had participated in. The KIDSCREEN scores of the two groups were compared using nonparametric statistics.

**Results:**

The questionnaires were completed by the parents of 67 children with SLI and 67 unaffected children. For children with SLI, the mean HRQOL scores were significantly lower for physical and psychological well-being, autonomy and parent relation, social support, and school environment compared to the reference group, controlling for age and parental education (β = -6.7 (-12.7;-.7) P = 0.03, β = -4.9 (-9.5;-.3) P = 0.04, β = -8.4 (-14.2;-2.6) P = 0.005, β = -11.6 (-19.5;-3.7) P = 0.004, β = -7.1(-12.4;-1.7) P = 0.010, respectively). Multivariate analyses in the group of children with SLI found that children who had undergone psychotherapy sessions or who had been enrolled in specific schooling programs had reduced HRQOL scores in social support and school environment and that children who were in a special class had higher scores in physical well-being.

**Conclusion:**

Children with SLI had significantly lower HRQOL scores as compared to unaffected children. Measurement of HRQOL could serve as one of the strategies employed throughout the follow-up of these individuals to provide them with the most appropriate and comprehensive care possible.

## Introduction

Specific language impairment (SLI) is a developmental language disorder characterized by impaired language acquisition from early childhood onward. It also manifests itself as problems in communication and learning. SLI is a common disorder, with a prevalence of 1–8% among kindergarten-age children and an incidence of 0.5–1% among the entire student population [1]. As defined by the standards set by the World Health Organization in the International Statistical Classification of Diseases and Related Health Problems in 2010, this disorder is not due to neurological or speech mechanism abnormalities, sensory impairment, intellectual disability, or environmental factors. Supporting children with SLI requires responsive health and education service models that can change and adapt to the child and young person’s needs [2]. The needs of these children could potentially be assessed by evaluating their health-related quality of life (HRQOL) and highlighting possible areas of weakness. On an individual level, the total HRQOL is defined by several dimensions including physical, mental, and social health perceptions (including health risks and conditions), functional status (mobility, autonomy, and dependency), social support, and socioeconomic status [3].

Analysis of HRQOL surveillance data could potentially help identify needs and guide interventions aimed at providing school-age children with SLI the most appropriate and comprehensive care possible [3]. However, the association between language impairments and the HRQOL in this population remains unclear; only a small number of studies have investigated the impact of language disorders on HRQOL in school-age children. Three large cohort studies performed in the Netherlands demonstrated that language impairment can have a substantial effect on the behavior and daily life of school-age children, as children with SLI have less favorable quality of life scores compared to those without this disorder, especially at a young age [4–6]. In contrast, a study in Finland found no discernible differences in the total HRQOL or in the individual dimension scores of children with SLI versus control subjects in the same 8 to 11 year age [7]. Three other studies with varied sample sizes suggested that despite a history of mild to severe SLI, adolescents and young adults with SLI did not differ significantly from control subjects in subjective perceptions of their quality of life [8–10]. These conflicting reports may be explained by the use of different measures of HRQOL used in each study (TACQOL [4], TAPQOL [5], CHQ-PF 28 [6], HRQOL 17D [7]), the variety of age categories (ranging from preschool children to adults), or differences in the educational and cultural systems of the countries studied. In light of the apparent lack of consensus regarding the HRQOL of children with SLI in the literature, no clear conclusion can be drawn from the current published data.

The KIDSCREEN-52 HRQOL questionnaire was the first HRQOL instrument developed via an international effort specifically for the evaluation of children or adolescents from 8 to 18 years of age and their parents [11]. Because of this international component and its validation in a large representative sample of children and adolescents, the KIDSCREEN questionnaire provides a broad perspective on understanding and interpreting HRQOL across different countries, therefore avoiding possible cultural biases regarding instrument content [12, 13]. A shorter version (the KIDSCREEN-27) was later developed, with 27 items grouped in five different dimensions comprising the total quality of life score: five items about physical well-being (“has your child felt fit and well?” or “in general, how would your child rate her/his health?”), seven items about psychological well-being (“has your child felt that life was enjoyable?” or “has your child been happy with the way he/she is?”), seven items about autonomy and parent relation (“has your child been able to do the things that he/she wants to do in his/her free time?” or “has your child felt that his/her parent(s) treated him/her fairly?”), four items about peers and social support (“has your child had fun with his/her friends?” or “has your child been able to rely on his/her friends?”), and four items about school environment (“has your child been happy at school?” or “has your child got along well with his/her teachers”) [13, 14]. Both the KIDSCREEN-52 and the KIDSCREEN-27 meet the criteria of a properly designed evaluation: measurable responses, high reliability, good validity, meaningful interpretability, alternative means of administration (self-/proxy report), and appropriate cultural adaptations [11, 12, 14–16]. The KIDSCREEN-27 reduces response burden and prevents missing data as it requires only 10 to 15 minutes to complete, compared to 15 to 20 minutes for the KIDSCREEN-52, but still permits evaluation of the main components of HRQOL [14].

The goal of this project was therefore to assess the HRQOL of children with SLI compared to their typically developing peers by the use of an appropriate HRQOL measure. Our study was designed to contribute additional information on the association between language impairments and HRQOL, and to investigate the effects of individual rehabilitation or school organization on the HRQOL of children and adolescents with SLI provided in our current systems of care. The aim of this study was to focus on children who reported reduced HRQOL to provide them targeted and early interventions in order to help them to develop strategies to outgrow their difficulties. In our mind, measurement of HRQOL could serve as one of the strategies that are used in the course of the follow-up of these individuals to provide them with the most appropriate and comprehensive care possible. In order to address some of the discrepancies in previous studies, we controlled for age and maternal education as potential confounding factors. Because HRQOL scores change with time, we confirmed that our results were not due to age differences between children with SLI and unaffected children. Maternal education [17], which is often used as a proxy for household income [18–20], was included as a second confounding factor as parental education beyond the minimum school-leaving age reduces the risk for persistent language problems [17, 21].

Our global hypothesis was that children and adolescents with SLI would show lower scores in all dimensions of HRQOL (physical well-being, psychological well-being, autonomy and parent relation, peers and social support, and school environment) compared to healthy children as measured by the different dimensions of the KIDSCREEN-27 evaluation. Specifically, we predicted lower scores on physical well-being based on the association between language impairment and developmental coordination disorder [22]. Twenty to 75 percent of children with SLI have motor difficulties, which impairs both motor and overall quality of life scores [4]. Additionally, low scores in psychological well-being could be expected as it is well known that developmental language difficulties affect mental health from school entry into adulthood [23]. Several studies have shown that children with SLI experience difficulty achieving independence in daily life [4, 24]. Moreover, parents of children with SLI usually experience more stress in the exercise of their parental roles than parents of healthy children which could affect the willingness of parents to support their child’s autonomy, therefore increasing parental dependence [6, 25]. According to the literature, language impairments in school-age children have a large impact on peer relations and are often related to poor social outcomes in childhood, adolescence, and even into adulthood [26–29]. We therefore expected impaired HRQOL in the domain of “peers and social support” due to the stigmatization of children with SLI [30, 31]. Finally, children with SLI were expected to have low scores related to “school environment” as language impairments negatively influence a child’s attitude towards school work and behavior towards others [6, 22] from the beginning of their schooling until the immediate post school years where they have been shown to have more academic difficulties and unemployment than controls [23, 32, 33].

## Methods

### Study design and population

The European KIDSCREEN group authorized the use of the French version of the KIDSCREEN-27 questionnaire, the only version used in this study.

The questionnaires were provided to 70 parents of SLI children and adolescents. Sixty-eight parents (i.e. 96%) completed and returned the questionnaires. A single uncompleted questionnaire was excluded from the study.

A further 67 questionnaires were completed by parents of typically developing children consulting in the pediatric emergency ward at Nantes Hospital. The response rate for the control group was 72%.

This study was a non-interventional research.

#### Participants with SLI

All children and adolescents between 8 and 18 years of age who were referred to the Learning Disabilities Reference Center in Nantes University Hospital (France) between January 1, 2014 and March 31, 2015 were included in the study sample. Learning Disabilities Reference Centers, created in the University Regional Hospitals following a ministerial decision in 2001 in France, are multidisciplinary teams made up of a pediatric neurologist, a neuropsychologist, a speech therapist, a psychomotor therapist, and a specialized teacher for communication-impaired children. These professionals perform tests in order to diagnose the language disorder, the severity, and the presence of other associated disorders. They are responsible for the coordination of care and service planning for children with language impairment. If warranted, they may also plan a second assessment test a few years later to adapt interventions to the children’s needs. Learning Disabilities Reference Centers use ICD-10 criteria to define specific language impairment. According to ICD-10, specific developmental disorders of language are disorders in which normal patterns of language acquisition are disturbed from the early stages of development.

In the Learning Disabilities Reference Centers, different tests were used to diagnose the impairment of language, according to the child’s age (S1 Table). Each test used standardized calibration and results were compared to the results of the children of the same age in France. All language skills fell more than two standard deviations below the mean according to the ICD-10 definition. IQ score was not an inclusion criterion because the IQ score includes non-verbal IQ and verbal IQ. As children with SLI have low scores in verbal IQ, they exhibit lower overall IQ scores compared to healthy children. Nevertheless, we confirmed using the Wechsler Intelligence Scale for Children that non-verbal IQ was above 70 to exclude children with intellectual disability [34]. Moreover, the children were deemed to have normal hearing, no obvious neurological deficits, and no known metabolic or genetic syndromes.

The characteristics of the specific language-impairment group were recorded, including the presence of other cognitive impairments, the number and length of rehabilitative interventions over time, specific schooling programs that had been provided to the afflicted child, as well as the degree to which they had been assigned a classroom assistant and/or been enrolled in special classes.

The questionnaires were distributed to the children’s parents at the conclusion of the consultation with the pediatric neurologist, and they were either completed on-site or subsequently mailed to the study coordinator.

#### Control subjects

Children between 8 and 18 years of age who were in general good health and who did not exhibit a history or signs of SLI were selected consecutively when referred to the Pediatric Emergency ward of Nantes University Hospital (France) between February 1, 2014 and May 1, 2014 for diagnosis and treatment of a first contusion or uncomplicated fracture. Exclusion criteria included repeated fractures, learning disorders with or without rehabilitation, and affliction with a chronic disease.

The questionnaires were distributed by the emergency physicians and were collected at the end of the consultation. Typically developing children were matched by age and gender to participants with SLI.

### Variables and measurement

For the purposes of this manuscript, we followed the previously established relevant guidelines, including all the factors considered to be important for incorporating HRQOL assessments into clinical trials [35]. All guidelines are available on the EQATOR Network’s website.

#### Demographic characteristics

Information regarding the age (in months) and gender (girls/boys) was provided by the parents. We also collected information about maternal education [17] and classified it into three categories: less than a bachelor’s degree, awarded a bachelor’s degree, and more than a bachelor’s degree.

#### Quality of life

The HRQOL of the children was assessed using the parent version of the KIDSCREEN-27 questionnaire. A 5-point Likert scale is used to rate individual items, with scores ranging from 1 (“not at all/never”) to 5 (“extremely/always”). The sum scales for each subscale were standardized to a scale ranging from 0 (worst) to 100 (best possible response pattern) because the five subscales differ in the number of items [13].

The European KIDSCREEN group authorized the use of the French version of the questionnaire, which was the only version used in this study.

For the overall sample, internal consistency (Cronbach’s α) ranged from 0.71 to 0.87 (0.71 for autonomy and parent relation, 0.73 for school environment and physical well-being, 0.75 for psychological well-being, and 0.87 for peers and social support).

#### Care of children and adolescents

The following data sets were collected from the parents and the medical records.

1. The presence of other cognitive impairments: reading and writing impairments, attention-deficit disorders, memory problems, motor disorders, and anxiety disorders.
2. The regularity and frequency of speech therapy and psychotherapy sessions based on historical arrangements. It was classified into four categories for each therapy: no sessions, rated 1; occasional isolated sessions, rated 2; less than or once a week regular sessions, rated 3; and more than once a week regular sessions, rated 4.
3. Specific school organization, based on current schooling in relation to specific schooling programs, having been assigned a classroom assistant, and having been enrolled in special classes. Specific schooling programs consisted of special language-based instruction in addition to regular education with occasional exam accommodation procedures or specialized instructional materials. Having specific schooling programs was recoded 1 and not having specific schooling programs was recoded 0. Having been assigned a classroom assistant was recoded 1 and the opposite situation was recoded 0. Schooling in special classes, defined by special education in special educational schools, was recoded 1. Not being schooled in special classes was recoded 0.

### Statistical analysis

Results obtained by Flapper et al. [4] revealed a lower overall quality of life mean score for the SLI group without developmental coordination disorders compared to the reference group (86.7 versus 90.5, respectively, which corresponds to a difference in the quality of life of 3.8 points between the two groups). Based on these results, we hypothesized that the difference in the HRQOL between children with SLI and healthy children would be at least 3.8-fold for each dimension of HRQOL after standardization to a scale ranging from 0 to 100, with a power of 90% and an alpha-risk of 5%. Based on these assumptions, a sample size of 64 would be required for each group.

All data were entered anonymously into an Excel spreadsheet. Questionnaires that had not been fully completed were not considered further. Non-parametric statistics were employed because of the non-normality of the distribution of variables, assessed by the Shapiro–Wilk test.

The KIDSCREEN scores of children with SLI were compared to those obtained from typically developing French control subjects using univariate analyses (Mann-Whitney tests) and multivariate analyses controlling for age and parental education. Multivariate analyses were multiple linear regressions. In the first model, dependent variables were KIDSCREEN scores (KIDSCREEN sub-scales) and independent variables were age, parental education and the presence or not of language impairment. In the second model, only children with SLI were included. Dependent variables were KIDSCREEN sub-scales and independent variables were rehabilitation sessions (speech therapy and psychotherapy), specific school organization (specific schooling programs, classroom assistant, and special classes), age and parental education. The statistical analyses were performed using the Stata statistical software package. Statistical significance was defined as P < 0.05.

### Ethical consideration

The Ethics Committee for Health in Nantes (GNDES) and the National Commission on Informatics and Liberty (CNIL) (No. 1748245v0) approved the study design. The participants and their parents received oral and written information about the study. We obtained verbal informed consent from the parents of the children enrolled in the study. There was no need to obtain written consent, according to GNDES, as the study was a non-interventional study. When verbal consent was obtained, we recorded consent by ticking the first box on the paper including inclusion criteria of children with SLI and healthy children.

## Results

### Study parameters

#### Study population

The characteristics of the two groups are shown in Table 1. No significant differences were discernible between children with SLI and typically developing children, except for the level of maternal education. The proportion of mothers who did not hold a university bachelor's degree was higher for the group of children with SLI (51%) than for the control group (25%).

**Table 1:** Demographic characteristics of the specific language-impairment (SLI) and control (C) groups. Mann-Whitney *U*-test to compare median scores and Chi-square test to compare percentage.

	SLI (*n* = 67)	C (*n* = 67)	Statistics test	P-value
Median age in months (Q1; Q3)	123 (108; 147)	134 (114; 157)	*u* = 1882	.11
Gender			χ^2^ = .496	.48
Girls n (%)	25 (37)	29 (43)		
Boys n (%)	42 (63)	38 (57)		
Level of maternal education			χ^2^ = 9.510	.009
Less than a bachelor’s degree n (%)	34 (51)	17 (25)		
Awarded a bachelor’s degree n (%)	11 (16)	20 (30)		
More than a bachelor’s degree n (%)	22 (33)	30 (45)		

#### Characteristics of children with SLI

Other cognitive impairments, individual rehabilitation, and specific school organization of the SLI group are summarized in Table 2.

**Table 2:** Characteristics of the specific language-impairment (SLI) group regarding other cognitive impairments, individual rehabilitation and specific school organization.

Variable		SLI (*n* = 67)
Other cognitive impairments *n* (%)		61 (91)
Reading and writing impairments		41 (61)
Attention-deficit disorders		9 (13)
Memory problems		18 (27)
Motor disorders		6 (9)
Anxiety disorders		29 (43)
Rehabilitation *n* (%)		
Speech therapy		
	No sessions	0
	Occasional isolated sessions	0
	Less or once a week regular sessions	22 (33)
	More than once a week regular sessions	45 (67)
Psychotherapy		
	No sessions	28 (42)
	Occasional isolated sessions	24 (36)
	Less or once a week regular sessions	15 (22)
	More than once a week regular sessions	0
Additional special education *n* (%)		
Specific schooling program		36 (54)
Classroom assistant		35 (52)
Special class		15 (22)

In our study, 91 percent of children with SLI reported other cognitive impairments including reading and writing impairments, attention-deficit disorders, memory problems, motor disorders and/or anxiety disorders. Among the 67 children with SLI, 29 children reported one other cognitive impairment and 32 children reported two or more other cognitive impairments.

Regarding individual rehabilitation, all children with SLI had regular speech therapy sessions and more than half of the children with SLI had psychotherapy sessions.

Moreover, more than half of the children with SLI attended specific schooling programs and had been assigned a classroom assistant, based on current schooling. Twenty-two percent of children were enrolled in special classes.

### Quality of life

The medians of each of the 27 items of the parental questionnaire for the children with SLI and the typically developing children are presented in Table 3.

**Table 3:** Detailed KIDSCREEN scores of the specific language-impairment (SLI) and control (C) groups.

Sub-scales KIDSCREEN-27	SLI (*n* = 67)	C (*n* = 67)
	Median	Median
Physical well-being		
1. Item 1. In general, how would your child rate her/his health?	100.00	100.00
2. Item 2. Has your child felt fit and well?	75.00	75.00
3. Item 3. Has your child been physically active (e.g. running, climbing, biking)?	50.00	75.00
4. Item 4. Has your child been able to run well?	75.00	75.00
5. Item 5. Has your child felt full of energy?	75.00	75.00
Psychological well-being		
1. Item 1. Has your child felt that life was enjoyable?	75.00	75.00
2. Item 2. Has your child been in a good moon?	75.00	75.00
3. Item 3. Has your child had fun?	75.00	75.00
4. Item 4. Has your child felt sad?	75.00	75.00
5. Item 5. Has your child felt so bad that he/she didn’t want to do anything?	100.00	100.00
6. Item 6. Has your child felt lonely?	100.00	100.00
7. Item 7. Has your child been happy with the way he/she is?	50.00	75.00
Autonomy and parent relationship		
1. Item 1. Has your child had enough time for him/herself?	50.00	75.00
2. Item 2. Has your child been able to do the things that he/she wants to do in his/her free time?	50.00	75.00
3. Item 3. Has your child felt that his/her parent(s) had enough time for him/her?	50.00	75.00
4. Item 4. Has your child felt that his/her parent(s) treated him/her fairly?	50.00	75.00
5. Item 5. Has your child been able to talk to his/her parent(s) when he/she wanted to?	75.00	75.00
6. Item 6. Has your child had enough money to do the same things as his/her friends?	50.00	50.00
7. Item 7. Has your child felt that he/she had enough money for his/her expenses?	00.00	25.00
Social support and significant others		
1. Item 1. Has your child spent time with his/her friends?	50.00	75.00
2. Item 2. Has your child had fun with his/her friends?	50.00	75.00
3. Item 3. Have your child and his/her friends helped each other?	50.00	75.00
4. Item 4. Has your child been able to rely on his/her friends?	50.00	75.00
School environment		
1. Item 1. Has your child been happy at school?	75.00	75.00
2. Item 2. Has your child got on well at school?	75.00	75.00
3. Item 3. Has your child been able to pay attention?	50.00	75.00
4. Item 4. Has your child got along well with his/her teachers?	75.00	75.00

The medians and ranges of the five sub-scales of HRQOL of the two groups are presented in Table 4. In univariate and multivariate analyses controlling for age and parental education, the medians were significantly higher for typically developing children on all dimensions of HRQOL (i.e. physical well-being, psychological well-being, autonomy and parent relation, peers and social support, and school environment).

**Table 4:** Comparison of the KIDSCREEN scores of the specific language-impairment (SLI) and control (C) groups.

Sub-scales KIDSCREEN-27	SLI (*n* = 67)		C (*n* = 67)		Univariate analyses		Multivariate analyses*		
	Median	Q1; Q3	Median	Q1; Q3	*u*	*p*	β	*p*	(95% CI)
Physical well-being	66.67	56; 78	72.22	61; 83	1665	0.010	-6.7	0.028	(-12.7;-.7)
Psychological well-being	75.00	61; 82	82.14	75; 86	1691	0.014	-4.9	0.039	(-9.5;-.3)
Autonomy and parent relation	50.00	39; 61	64.29	46; 75	1511	0.001	-8.4	0.005	(-14.2;-2.6)
Peers and social support	50.00	36; 69	68.75	50; 81	1509	0.001	-11.6	0.004	(-19.5;-3.7)
School environment	62.50	56; 75	75.00	56; 81	1700	0.015	-7.1	0.010	(-12.4;-1.7)

*reference for multivariate analyses, controlling for age and parental education**:** healthy control

### Predictors of quality of life for children with SLI

Analysis of the data from the questionnaires for associations between rehabilitation, specific school organization, and dimensions of HRQOL revealed that only four correlations reached statistical significance (Table 5). Children with SLI who had undergone psychotherapy, both occasional and regular sessions, had reduced parent-reported scores in “peers and social support.” In contrast, the frequency of speech therapy sessions were not associated with change in any HRQOL scores. Children who had been enrolled in specific schooling programs had reduced scores in the “school environment.” Children who were in a special class had higher scores in “physical well-being.” The presence of a classroom assistant to help the child was not associated with any change in HRQOL scores.

**Table 5:** Multivariate analyses on KIDSCREEN in specific language-impairment (SLI) group, controlling for age and parental education.

	SLI (*n* = 67)														
	Physical well-being			Psychological well-being			Autonomy and parent relation			Peers and social support			School environment		
	β	*p*	(95% CI)	β	*p*	(95% CI)	β	*p*	(95% CI)	β	*p*	(95% CI)	β	*p*	(95% CI)
Rehabilitation															
Speech therapy*															
More than once a week regular sessions	-3.9	0.43	(-13.9;6.1)	0.9	0.82	(-7.2;9.1)	-4.3	0.32	(-13.0;4.4)	-1.0	0.87	(-12.5;10.5)	4.9	0.20	(-2.7;12.4)
Psychotherapy**															
Occasional isolated sessions	-5.9	0.26	(-16.2;4.4)	-4.6	0.28	(-13.0;3.8)	-2.7	0.54	(-11.7;6.2)	-14.1	0.02	(-26.0;-2.3)	3.8	0.33	(-4.0;11.5)
Once a week or less regular sessions	0.9	0.87	(-10.7;12.6)	-3.2	0.50	(-12.7;6.3)	-0.9	0.85	(-11.0;9.2)	-15.1	0.03	(-28.5;-1.7)	3.9	0.38	(-4.9;12.6)
Specific school organization															
Specific schooling programs	2.6	0.58	(-6.9;12.1)	1.2	0.75	(-6.5;9.0)	1.3	0.76	(-7.0;9.5)	5.1	0.35	(-5.8;16.0)	-7.4	0.04	(-14.6;-.3)
Classroom assistant	0.9	0.86	(-8.8;10.5)	-1.5	0.71	(-9.4;6.4)	2.9	0.50	(-5.5;11.2)	6.8	-0.23	(-17.9;4.3)	2.6	0.54	(-4.7;9.6)
Special classes	12.0	0.03	(-1.2;22.9)	1.7	0.70	(-7.1;10.5)	-1.5	0.75	(-10.9;7.8)	2.1	0.73	(-10.3;14.6)	2.5	0.54	(-5.6;10.7)

*reference for speech therapy: once a week or less regular sessions

**reference for psychotherapy: no session

## Discussion

We investigated the HRQOL of a clinical sample of children between 8 and 18 years of age who had been diagnosed with SLI. According to the data provided by the children’s parents, the children with SLI had a lower HRQOL than typically developing children in all dimensions: physical and psychological well-being, autonomy and parent relation, peers and social support, and school environment. Only a few associations between rehabilitation, specific school organization, and dimensions of HRQOL were found. Lower scores in social support as well as school environment were more frequent among the children with SLI who had undergone psychotherapy sessions or who had been enrolled in specific schooling programs. Children who were in a special class had higher scores in physical well-being.

In this study we clearly demonstrated that language problems have an impact on children’s HRQOL. Children with SLI differ from their peers in terms of general well-being and autonomy. They seem to be less energetic and less active than typically developing children. Moreover, parents tend to state that children with SLI may have a poor understanding of others and consequently, they are less capable of playing with other children, are less at ease, and are less sure of themselves when in the company of others [5]. As they progress through the school system, adolescents with SLI are at risk of becoming targets for victimization, and this can adversely impact their social integration [30]. Apart from communication problems which may explain the lower HRQOL for children with SLI in the social domain, other common co-occurring learning disorders could play a role in lowering a child’s HRQOL as we have seen in this study and in literature [4, 36, 37]. All these findings are in agreement with those of other studies regarding the HRQOL of children with SLI [4–6, 30]. In contrast, three studies have shown that adolescents and young adults with SLI do not differ from their peers in terms of personal happiness and life satisfaction [8–10]. We postulate that, unlike children who tend to have difficulty adapting to such learning disorders, adolescents and young adults with SLI develop coping strategies, often in conjunction with rehabilitation.

More than half of the children in the SLI group received psychotherapy, either through occasional or regular sessions. As reported in medical records and the literature, various symptoms lead to children being referred for psychotherapy: lack of self-confidence, shyness, depressive and anxious symptoms, immature behavior, impulsiveness, attention deficit, hyperactivity, mood management difficulties, or sleep disorder [27, 38–40]. In our study, children with SLI who were reported to receive psychotherapy sessions were more likely to have reduced HRQOL in the dimension of peers and social support. It could very well be that children and adolescents with SLI who attend psychotherapy sessions have more social, psychological, or personality challenges to begin with. This means that not only do they have to deal with language impairments, they also have to cope with psychological difficulties which could be a real handicap in daily life. This could potentially explain the lower HRQOL scores in the “peers and social support” dimension [27, 39]. Moreover, the association between psychotherapy sessions and low HRQOL scores in “peers and social support” may be explained in part by results of the study of Conti-Ramsden and Botting, who showed that children with SLI were at risk of being targets for victimization and often experienced poor social outcomes [30]. These conclusions indicate that children with SLI need regular psychotherapy sessions to be self-confident, adapt themselves to peers, and improve their social quality of life.

Interestingly, our results did not reveal any relationship between the frequency of speech therapy sessions and HRQOL scores. This lack of association could be explained by the fact that the children with SLI who needed regular and frequent speech therapy sessions were likely to be those with the most severe language impairments and consequently to be those who would have reported the lowest HRQOL scores without rehabilitation. In these cases, intensive speech therapy may have improved the HRQOL scores of severely affected children to equal those of children with moderate language impairments. On the other hand, the lack of association between the frequency of speech therapy sessions and HRQOL scores may be due to a lack of statistical power of the study as sample sizes had been determined using the main hypothesis, which was the difference in the HRQOL between children with SLI and healthy children. As the children with SLI enrolled in regular and frequent speech therapy sessions represents a subset of our total cohort, a larger sample may be required to show an association between rehabilitation and HRQOL scores.

In our study, children with SLI who have been enrolled in specific schooling programs showed reduced HRQOL scores in the school environment. This may be explained by the fact that children receiving specific schooling programs are likely to be children with more severe language and social difficulties. They may have been supported later, as the identification and implementation of schooling programs is a process that takes time. Moreover, schooling programs may not be adapted to the child initially at school entry, which could explain the reduced HRQOL scores. Consequently, these programs have to be regularly adjusted according to each child’s needs and should be discussed and reassessed by multidisciplinary teams in Learning Disabilities Reference Centers.

Surprisingly, children with SLI enrolled in special classes, had higher physical well-being than children with SLI in regular educational school. It is well known that children with SLI often have motor co-morbidity, such as praxic difficulties like imitation or planning difficulties, and/or fine and gross motor difficulties [4, 22, 37]. Special classes consist of special education in restrictive settings, which imply that the number of children in each class is reduced and teaching is adapted to each child’s abilities. In special classes, sports exercises and games are likely to be adapted to each child and may be focused on the physical well-being of children. Moreover, rehabilitation is usually included as a component in special educational schooling. It could be that children with SLI in special classes have more rehabilitation sessions, such as physical therapy, psychomotricity sessions, and/or occupational therapy which could improve their physical well-being. To conclude, we recommend that specific school organization and appropriate therapy sessions be implemented for children with severe language disabilities in order to adjust the children’s schooling accordingly and cope with their language disadvantages.

Our study is not without its limitations, such as the fact that the HRQOL was measured indirectly from the data provided by the parents. Proxy measures can induce judgment biases, and it is generally accepted that HRQOL reported by parents are often lower than HRQOL reported by the children themselves [41]. Self-reports were not part of the planned study procedures for this initial investigation based on French subjects; however, we are fully cognizant that this aspect is an important issue that should be taken into account in future studies. As children with severe language impairment may experience difficulties with interpreting and answering questions, it might be appropriate to use specifically tailored questionnaires for children that use pictures or comics. In future studies, it will be interesting to compare the individual responses made by parents to those made by the children themselves and to evaluate the influence of different points of view on HRQOL scores. Another limitation of our study is that we used standardized HRQOL scores, as per a previous study about HRQOL among children with mental health problems published in 2012 [42], instead of the Rash-scaled HRQOL which are difficult to analyze and interpret for readers.

## Supporting Information

S1 Table Language tests in specific language impairment group. (DOC)

## Abbreviations

SLI: Specific language impairment
HRQOL: Health-related quality of life
