# Supplementary material for: Health-Related Quality of Life for Children and Adolescents with Specific Language Impairment: A Cohort Study by a Learning Disabilities Reference Center
Source: PLoS One. 2016 Nov 16;11(11):e0166541. doi: 10.1371/journal.pone.0166541 (PMC5112866; doi:10.1371/journal.pone.0166541)
Supplement: S1 Table — (DOC) [file pone.0166541.s001.doc]

**S1 Table. Language tests in specific language impairment group.**

|  | Children between 8 to 11 years of age | Children between 11 to 15 years of age |
| --- | --- | --- |
| Receptive language tests |  |  |
| Lexical skills | Picture vocabulary scale (EVIP) | Picture vocabulary scale (EVIP) |
| Morpho-syntactic comprehension | Test of syntactic and semantic comprehension (ECOSSE) | Test of syntactic comprehension (TCS) |
|  |  |  |
| Expressive language tests |  |  |
| Phonology | Screening tool dyslexia (ODEDYS) | Computerized cognitive battery for teenagers aged 11 to 15 years (EXALANG 11-15) |
| Lexical skills | Battery for the evaluation of oral language, written language, memory and attentional skills (L2MA2) | Computerized cognitive battery for teenagers aged 11 to 15 years (EXALANG 11-15) |
| Morpho-syntactic expression | Battery for the evaluation of oral language, written language, memory and attentional skills (L2MA2) | Complex oral language test for colleges (TLOCC) |
| Speech informativeness | Battery for the evaluation of oral language, written language, memory and attentional skills (L2MA2) | Assessment protocol developed language for teenagers (PELEA) |
